# Effect of Contralateral Medial Olivocochlear Feedback on Perceptual Estimates of Cochlear Gain and Compression

**DOI:** 10.1007/s10162-016-0574-8

**Published:** 2016-08-22

**Authors:** Mark D. Fletcher, Katrin Krumbholz, Jessica de Boer

**Affiliations:** 1 Medical Research Council Institute of Hearing Research, The University of Nottingham, University Park, Nottingham, NG7 2RD UK; 2 School of Psychology, University of Nottingham, University Park, Nottingham, NG7 2RD UK; 3 Institute of Sound and Vibration Research, University of Southampton, Highfield Campus, Southampton, SO17 1BJ UK

**Keywords:** medial olivocochlear reflex (MOCR), temporal masking curve (TMC), click-evoked otoacoustic emissions (CEOAEs), contralateral acoustic stimulation, cochlear amplification

## Abstract

The active cochlear mechanism amplifies responses to low-intensity sounds, compresses the range of input sound intensities to a smaller output range, and increases cochlear frequency selectivity. The gain of the active mechanism can be modulated by the medial olivocochlear (MOC) efferent system, creating the possibility of top-down control at the earliest level of auditory processing. In humans, MOC function has mostly been measured by the suppression of otoacoustic emissions (OAEs), typically as a result of MOC activation by a contralateral elicitor sound. The exact relationship between OAE suppression and cochlear gain reduction, however, remains unclear. Here, we measured the effect of a contralateral MOC elicitor on perceptual estimates of cochlear gain and compression, obtained using the established temporal masking curve (TMC) method. The measurements were taken at a signal frequency of 2 kHz and compared with measurements of click-evoked OAE suppression. The elicitor was a broadband noise, set to a sound pressure level of 54 dB to avoid triggering the middle ear muscle reflex. Despite its low level, the elicitor had a significant effect on the TMCs, consistent with a reduction in cochlear gain. The amount of gain reduction was estimated as 4.4 dB on average, corresponding to around 18 % of the without-elicitor gain. As a result, the compression exponent increased from 0.18 to 0.27.

## **INTRODUCTION**

Animal data suggest that the medial olivocochlear (MOC) efferent reflex can exert powerful modulation of peripheral auditory responses, reducing the gain of the active cochlear amplifier (provided by the outer hair cells (OHCs)) by as much as 20–30 dB (Murugasu and Russell 1996). However, the function of the MOC reflex remains unclear. Animal research has suggested that the MOC reflex is frequency-specific (i.e., can only be elicited by a limited range of frequencies around the probe frequency; Warren and Liberman 1989) and primarily affects frequencies above those affected by the middle ear muscle (MEM) reflex (Guinan and Gifford 1988). This suggests that the MOC reflex may complement the MEM reflex to form a complete barrier against noise insult (Liberman and Guinan 1998). However, studies that have measured MOC-induced suppression of otoacoustic emissions (OAEs) in humans have yielded results that seem to contrast with the animal results, suggesting that the human MOC reflex mainly affects low frequencies, similar to those affected by the MEM reflex (Lilaonitkul and Guinan 2009a, 2012; Zhao and Dhar 2012), and is largely unspecific in frequency, at least when elicited by contralateral sounds (Lilaonitkul and Guinan 2009a, b, 2012; Zhao and Dhar 2012). Ipsilateral elicitor effects on OAEs have been found to be more frequency-specific (Lilaonitkul and Guinan 2009a, b, 2012), but it remains unclear, to what degree these effects are caused by intrinsic cochlear (non-efferent) mechanisms (Guinan et al. 2003).

The aim of the current study was to develop a reliable procedure for measuring the contralateral MOC reflex psychophysically in humans. Such a procedure would provide an independent measure of human MOC reflex properties, which could be compared with the previous OAE and animal results. Previous studies have suggested that the MOC reflex causes the psychophysical “overshoot” phenomenon, whereby the masked detection threshold of a short signal presented at the masker onset is lowered by a preceding “precursor” sound (Schmidt and Zwicker 1991; von Klitzing and Kohlrausch 1994; Strickland 2001, 2004, 2008; Strickland and Krishnan 2005). However, overshoot is not a suitable approach for measuring contralateral MOC function, because (i) MOC involvement in overshoot has been questioned (Bacon and Moore 1987; Scharf et al. 2008; Fletcher et al. 2015) and (ii) contralateral precursor effects have been hard to find (Bacon and Healy 2000; Bacon and Liu 2000). Several previous studies have measured contralateral MOC elicitor effects on psychophysical measures of cochlear frequency selectivity (Kawase et al. 2000; Quaranta et al. 2005; Vinay and Moore 2008; Aguilar et al. 2013; Wicher 2013; Wicher and Moore 2014). The active cochlear amplifier enhances cochlear frequency selectivity (Robles and Ruggero 2001), and so, MOC-induced reduction in amplifier gain should be associated with a decrease in frequency selectivity. Whilst the previous results have generally been consistent with this expectation, the observed effects have been weak (sometimes non-significant; Quaranta et al. 2005), and the pattern of results has been variable across studies (reviewed in Wicher and Moore 2014). Another approach, namely, to measure MOC elicitor effects on psychophysical estimates of cochlear gain and compression, has promised to yield more reliable and consistent results (Krull and Strickland 2008; Jennings et al. 2009; Roverud and Strickland 2010; Yasin et al. 2014). So far, however, this approach has only been applied with ipsilateral elicitors. Here, we used contralateral elicitors to eliminate the possibility of effects caused by non-efferent mechanisms.

**FIG. 1:**
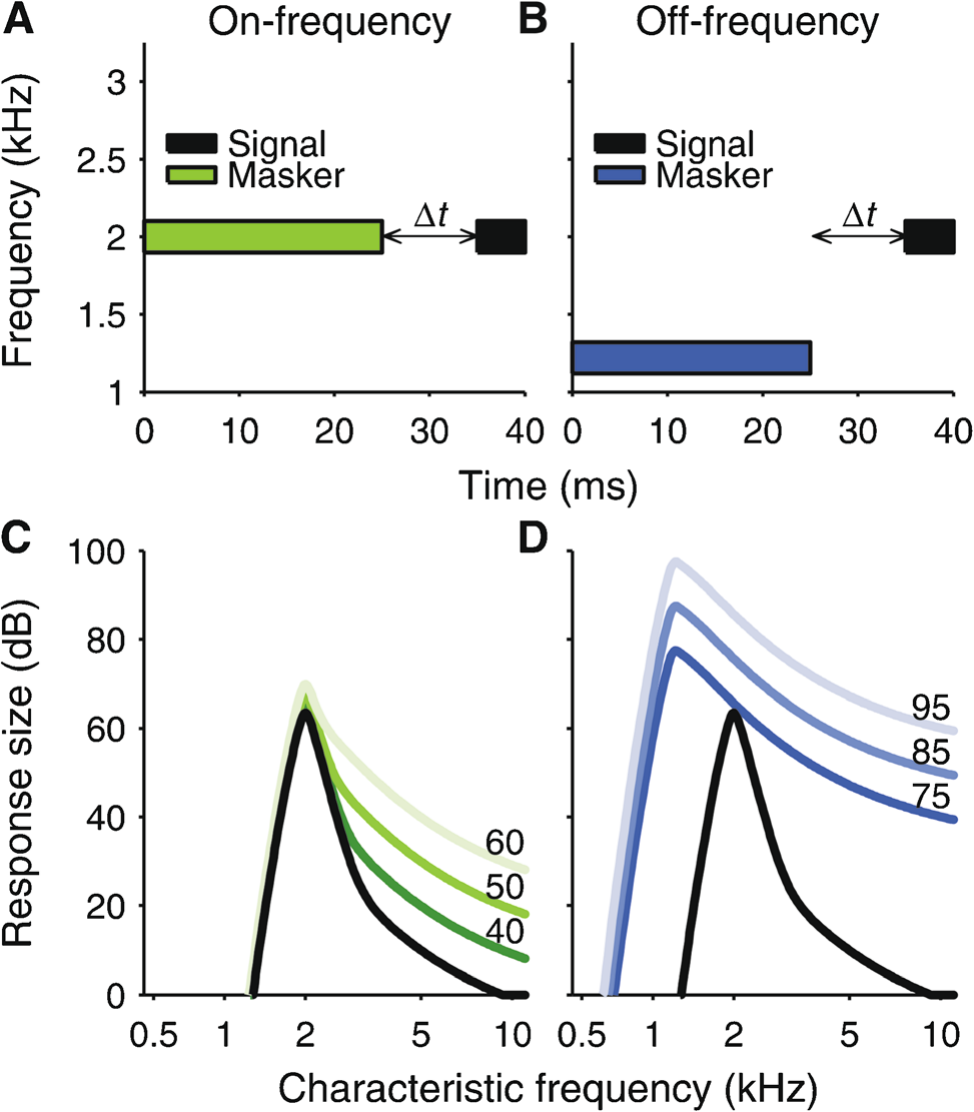
**A**, **B** Schematic representation of spectral and temporal stimulus characteristics for on- **(A)** and off-frequency **(B)** temporal masking curve (TMC) measurements. *Δt*: masker-signal gap. **C**, **D** Cochlear response patterns (“excitation patterns”) of the signal (*black line*) and the on- and off-frequency maskers (*green- and blue-shaded lines* in *panels* **C** and **D**, respectively). The patterns were calculated by modeling the cochlear filters as two rounded exponential functions (Patterson and Nimmo-Smith 1980), one representing the active tip filter and the other to the passive tail filter. The tip filters had an equivalent rectangular bandwidth (ERB) of ERB_*n*_ = 24.67 ⋅ (4.37 ⋅ CF + 1) Hz, where CF is the characteristic frequency in kilohertz (Glasberg and Moore 1990), and were centered at CF. The tail filters were centered a quarter octave below CF and their ERB was equal to 3 ⋅ ERB_*n*_. The tip filters had a gain of *G*(*L*) = max(min((*c* − 1) ⋅ (*L* − *BP*_1_) + *G*_max_, *G*_max_), 0), where *L* is the stimulus level, and *c* and BP_1_ are the compression exponent and lower edge of the compressive range of the tip response IO function, respectively (equal to 0.2 and 28.125 dB SPL in this simulation). The tail filters had zero gain. The signal was set to a level of 30 dB SPL (similar to the average signal level in the current experiment). The maskers were set to three different levels (shown in dB SPL to the right of the patterns).

The elicitor was a broadband noise, because contralateral sounds with broader bandwidths have been shown to be more effective MOC elicitors (Berlin et al. 1993; Maison et al. 2000; Lisowska et al. 2002; Velenovsky and Glattke 2002; Lilaonitkul and Guinan 2009a; Wicher and Moore 2014). Cochlear gain and compression were estimated using the “temporal masking curve” (TMC) method (Nelson et al. 2001), which measures forward-masking thresholds for a short sinusoidal signal as a function of the masker-signal gap (the function relating the masking threshold with the masker-signal gap is referred to as TMC; Fig. 1A, B). The signal is presented at a low level above the quiet threshold to create a well-localized response along the cochlear partition (Fig. 1C, D). When the masker is at the signal frequency (on-frequency condition; Fig. 1A), masking is caused by the tip of its cochlear response (Fig. 1C), which is subject to cochlear gain (and thus compression). In contrast, when the masker frequency is well below the signal frequency (off-frequency condition; Fig. 1B), masking is caused by the basal (high-frequency) tail of its response (Fig. 1D), which is not or little affected by gain (Robles and Ruggero 2001). The difference between the on- and off-frequency masking thresholds thus reflects the gain of the on-frequency masker tip response. The measurements were conducted at a signal frequency of 2 kHz, because this frequency has also been used in many of the previous measurements of contralateral elicitor effects on perceptual frequency selectivity (reviewed in Wicher and Moore 2014). Despite using a low elicitor level (to avoid eliciting the MEM reflex), we found a sizeable and reliable elicitor effect on the TMCs, consistent with a reduction in cochlear gain. The effect was comparable in size to that found previously in auditory nerve responses. The elicitor effect on the TMCs is compared with the effect on click-evoked OAEs (CEOAEs).

## **EXPERIMENT 1: CONTRALATERAL ELICITOR EFFECTS ON TEMPORAL MASKING CURVES**

Forward-masking thresholds were measured for a 5-ms, 2-kHz sinusoidal signal with and without a contralateral wideband-noise elicitor. The signal level was set at 10 dB above the individual thresholds in quiet (sensation level (SL)) and fixed across the with- and without-elicitor conditions. The masker was also sinusoidal, with a frequency either at (2 kHz), or 0.71 octaves below (1.22 kHz), the signal frequency (on-/off-frequency conditions; Fig. 1A, B). The masker duration (25 ms) was much shorter than in the original TMC paradigm (Nelson et al. 2001) to minimize the possibility for the masker itself to elicit the MOC reflex. The elicitor was presented at 54 dB sound pressure level (SPL), well below the lowest typanometric MEM reflex threshold across individuals. In the following, we describe expectations for the elicitor effect on the on- and off-frequency masking thresholds.

**FIG. 2:**
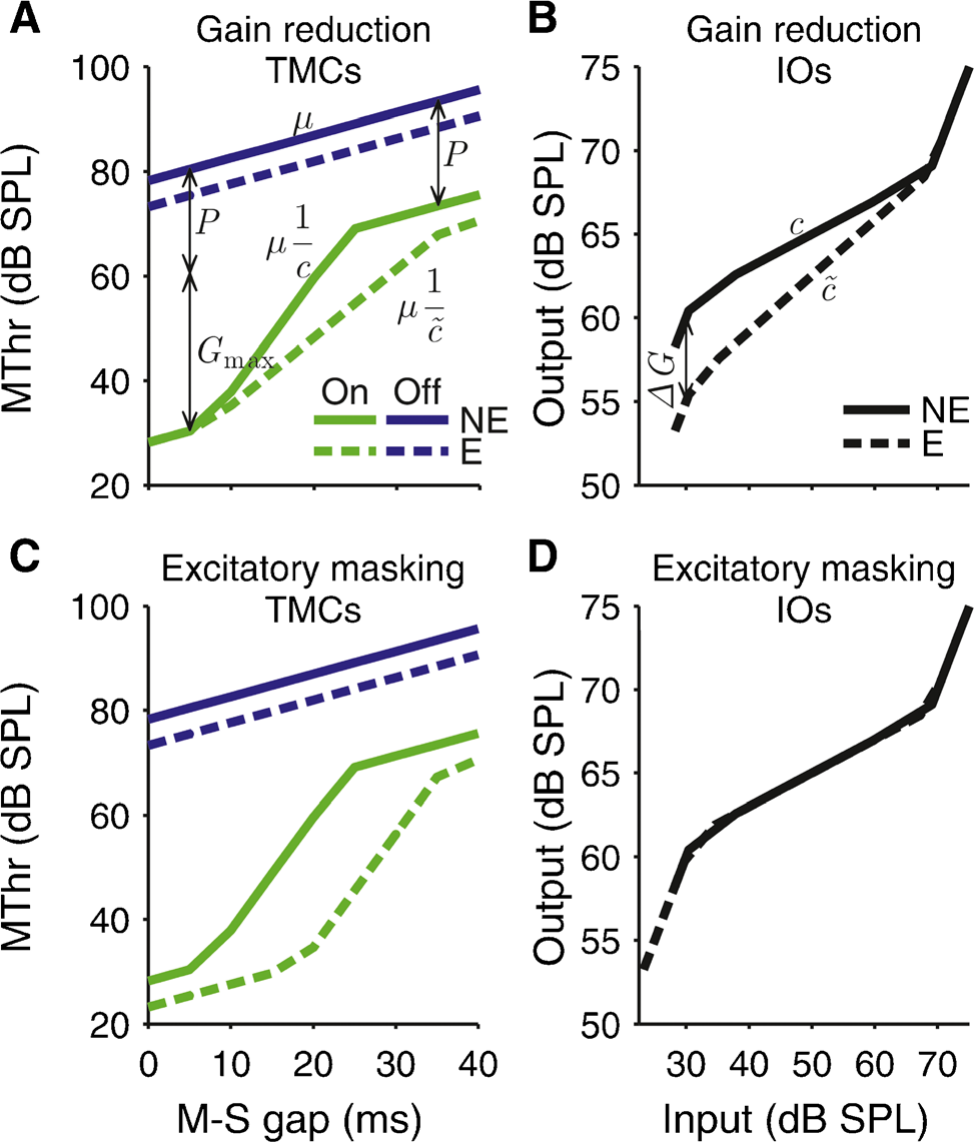
**A**, **C** Simulated on- and off-frequency TMCs (*green and blue lines*) without (*solid lines*) and with (*dashed lines*) an elicitor (labelled *NE* and *E*; see legend in **A**). For **A**, the elicitor was assumed to cause a reduction in cochlear gain, and for **C**, it was assumed to cause direct excitatory masking. Panels **B** and **D** show the respective inferred cochlear input-output (IO) functions (off- versus on-frequency masking thresholds minus passive attenuation of the off-frequency masker response, *P*). *G*_max_, *c*: maximum gain and compression exponent without the elicitor; *ΔG*, $\tilde{c}$: elicitor-induced gain reduction and with-elicitor compression exponent; *μ*: decay rate of masker effect.

The TMC method assumes that the rate of decay of the masker effect over time is independent of the masker frequency (Nelson et al. 2001). If the masker effect (in intensity units) decays exponentially with a rate *μ*, the off-frequency masker level required to mask the signal (in logarithmic decibel units) should increase linearly with increasing masker-signal gap, and the rate of increase should be equal to *μ* (solid blue line in Fig. 2A). Active cochlear amplification is maximal at low sound levels and is progressively disabled towards higher levels (Robles and Ruggero 2001). As a result, the input-output (IO) function of the on-frequency masker tip response will grow linearly (with a slope of unity) at low and high masker levels, but compressively (with a slope, *c*, less than unity) at intermediate levels (solid black line in Fig. 2B). As a result, the on-frequency masking threshold will grow linearly with a slope *μ* at short and long masker-signal gaps (like the off-frequency threshold), but at intermediate gaps, the slope will be steeper by a factor of $1/c$ (reciprocal of IO function slope; solid green line in Fig. 2A). At short masker-signal gaps, the difference between the on- and off-frequency thresholds should correspond to the sum of the active gain of the on-frequency masker tip response (*G*_max_ in Fig. 2A) and any difference between the on-frequency tip and off-frequency tail responses due to passive filtering (*P* in Fig. 2A). Towards longer masker-signal gaps, the difference decreases to the passive difference only.

If the elicitor causes a reduction in cochlear gain, the off-frequency threshold should decrease equally across all masker-signal gaps, by an amount corresponding to the gain reduction, *ΔG* (compare solid and dashed blue lines in Fig. 2A). This is because the signal response would be diminished by *ΔG*, but the off-frequency masker tail response would be unchanged. For the on-frequency condition, the effect of a gain reduction should depend on the masker-signal gap. At short gaps, a gain reduction should affect the signal and masker responses equally, and so, there should be no change in masking threshold (compare solid and dashed green lines in Fig. 2A). At intermediate gaps, a gain reduction should decrease the compressiveness of the on-frequency masker tip response (to $\tilde{c}>c$; compare solid and dashed lines in Fig. 2B), leading the on-frequency masking threshold to increase more shallowly (by a factor of $c/\overset{\sim }{c}$; Fig. 2A). At long masker-signal gaps, the difference between the on-frequency thresholds with and without the elicitor should, like the difference between the off-frequency thresholds, become equal to *ΔG*. The on- and off-frequency TMCs can be used to infer the IO function of the on-frequency masker tip response by plotting the off-frequency masking threshold for each masker-signal gap against the corresponding on-frequency threshold and correcting for the passive difference between the on- and off-frequency masker responses, *P* (Fig. 2B). If the elicitor causes a gain reduction, *ΔG*, the lower leg of the inferred IO function for the with-elicitor condition should be shifted downwards (towards lower output levels) by *ΔG*, and the IO function slope should become less compressive ($\tilde{c}$ > *c*; compare solid and dashed lines in Fig. 2B).

In addition to reducing cochlear gain, the elicitor may cause masking by “swamping” neurons responsive to the signal (referred to as excitatory masking; Delgutte 1990). With contralateral elicitors, any excitatory masking would be assumed to occur in central neurons responsive to both ears (Zwislocki 1972). Contralateral masking effects in electrically stimulated ears support this premise (James et al. 2001; Lin et al. 2013). Excitatory masking by the elicitor would be equivalent to a reduction in the signal response and should shift the on- and off-frequency masking thresholds towards longer masker-signal gaps by the same amount (Fig. 2C). As a result, the off-frequency threshold would decrease equally at all masker-signal gaps. Thus, in the off-frequency condition, the effect of excitatory masking would be indistinguishable from the effect of gain reduction (compare blue lines in Fig. 2A and C). In contrast, in the on-frequency condition, the effects of excitatory masking and gain reduction would be distinct; excitatory masking would reduce the on-frequency masking threshold even at the shortest masker-signal gap (by the same amount as the off-frequency threshold) and would leave the slope of the on-frequency TMC unchanged. As a result, the inferred IO function would also remain unchanged (Fig. 2D). Given that the off-frequency TMC does not distinguish between excitatory masking and gain reduction effects, it should be sufficient to measure only the on-frequency TMC with and without the elicitor and to measure the off-frequency TMC only without the elicitor. This was tested in a subset of subjects.

Results by Micheyl and Collet (1996) suggest that the effect of a contralateral elicitor may depend on the order in which the with- and without-elicitor conditions are measured. They found a correlation between contralateral OAE suppression and elicitor-induced improvement in signal-in-noise detection when the with-elicitor conditions preceded the without-elicitor conditions, but not vice versa. To control for any effects of the condition order, the on-frequency TMCs with and without the elicitor were either measured in separate sessions conducted on different days or interleaved within the same session.

### Methods

#### Subjects

A total of 12 subjects (7 males, aged between 20 and 31 years) participated in this study. Six (S1–S6; 3 males, aged between 20 and 26 years) measured both the on- and off-frequency TMCs with and without the elicitor. The other six (S7–S12; 4 males, aged between 20 and 31 years) only measured a reduced set of conditions, excluding the with-elicitor off-frequency TMC. One subject (S2) took part in the piloting.

Subjects were screened for normal hearing at audiometric frequencies between 0.25 and 6 kHz (absolute thresholds <20 dB HL) and for normal tympanometric peak pressure (−50–50 daPa) and compliance (0.3–1.6 ml; GSI TympStar, Grason-Stadler, Eden Prairie, MN, USA). They reported no history of audiological or neurological disease or use of neuroactive medication. They gave written informed consent and received an inconvenience allowance. The experimental procedures were approved by the Ethics Committee of the University of Nottingham School of Psychology and conformed to the guidelines of the Declaration of Helsinki at the time the data were collected (version 6, 2008), but were not formally pre-registered online in accordance with the 2014 amendment to the Declaration.

#### Experimental Protocol and Procedure

All thresholds were measured using a three-interval, three-alternative forced-choice adaptive tracking procedure. For the signal quiet thresholds (needed to set the signal level for the TMC measurements), one interval, chosen randomly with equal a priori probability, contained the signal, and the other two contained silence. For the masking thresholds (TMCs), one interval contained the signal and masker and the other two contained the masker only. The intervals were cued visually and separated by 500-ms gaps. The task was to identify the signal interval by pressing an appropriate response button. Visual feedback was given after each trial. The adaptive parameter was either the signal (signal quiet thresholds) or masker level (masking thresholds). The signal level was varied according to a two-down, one-up, and the masker level according to a two-up, one-down, procedure, which track 70.7 % correct performance (Levitt 1971). The step size was 10 dB up to the first reversal, 5 dB up to the second reversal, and 2.5 dB for the remaining ten reversals. Each track lasted about 2 min. The resulting threshold was estimated as the average of the last ten reversals. Six threshold estimates were acquired for each condition, and the set of three or more estimates with the minimum standard error averaged to obtain a final threshold estimate. Masking threshold estimates for different masker-signal gaps were acquired in a random order. At least 2 h of practice were given before data collection was started.

The on-frequency TMCs with and without the elicitor were measured either separately, in different sessions, or interleaved within the same session. In the interleaved sessions, threshold tracks with and without the elicitor were alternated. The off-frequency conditions were measured in a separate session, with alternating with- and without-elicitor tracks (if the with-elicitor off-frequency condition was measured). Different sessions were conducted on different days. The session order (separate, interleaved, off-frequency) was counter-balanced across subjects.

Each subject’s MEM reflex threshold was measured for a broadband (0.125–4 kHz) white noise. The noise was presented to the same ear as the elicitor (left) and the reflex threshold measured in the opposite (right) ear. The measurements were conducted with a GSI TympStar tympanometer. A reflex was taken as a change in the middle ear compliance of at least 0.02 ml.

#### Stimuli

The signal and maskers were sinusoids, presented to the right ear, and the elicitor was a broadband noise, presented to the left ear. The signal and on-frequency masker had a frequency of 2 kHz. The off-frequency masker had a frequency of 1.22 kHz, 0.71 octaves, or four normal auditory filter bandwidths (in units of equivalent rectangular bandwidth (ERB_N_); Glasberg and Moore 1990) below the signal frequency. Results by Lopez-Poveda et al. (2003) suggest that, at four ERBs below the signal frequency, the off-frequency masker tail response is little affected by cochlear gain. The elicitor was band-pass-filtered to a range of 20 ERB_N_ around the signal frequency (0.531–6.308 kHz). Within its passband, it was filtered to elicit equal energy per ERB_N_. The filtering was conducted in the frequency domain using a 2^19^-point fast Fourier transform (FFT) to create a 21.475-s cyclical noise buffer, which was played continuously throughout each threshold track. The band-pass filter was implemented as a boxcar. All stimuli were gated on and off with 5-ms quarter-sine and -cosine ramps, respectively. The signal and masker durations, measured between the −3-dB points, were 5 and 25 ms, respectively (corresponding to steady-state durations of 0 and 20 ms). The masker-signal gap (between the −3-dB points) was varied between 5 and 30 ms in 5-ms steps (corresponding to 0–25 ms between the 0-V points). Not all masker-signal gaps were measured in all subjects. The signal was presented at a level of 10 dB SL (fixed across the with- and without-elicitor conditions). The masker level was varied adaptively. The elicitor was presented at a level of 40 dB per ERB_N_ (54 dB SPL overall).

All stimuli were generated digitally at a sampling rate of nearly 25 kHz using TDT System 3 (Tucker-Davies Technologies, Alachua, FL, USA) and MATLAB (The Mathworks, Natick, MA, USA). They were digital-to-analogue converted with a 24-bit amplitude resolution (TDT RP2), amplified (TDT HB7), and presented through Sennheiser HD 600 headphones (Wedemark-Wennebostel, Germany) in a double-walled, sound-attenuating booth (IAC, Winchester, UK).

#### Statistical Analysis

Individual masking thresholds were analyzed with multilevel linear models (MLMs), implemented in *R* (R Core Team 2012) using the *lmer* function, which is part of the *lme4* package (Bates et al. 2013). The effect of masker-signal gap was modelled as fixed linear and quadratic covariates. The quadratic covariate was included to account for the non-linear shape of the on-frequency TMCs. The effects of masking condition (on/off), elicitor condition (with/without), and session type (separate/interleaved) were included as fixed factors. The models also included by-subject random intercepts and by-subject random slopes for the linear masker-signal gap covariate. Where appropriate, additional by-subject random slopes for the effects of masking condition, elicitor condition, or the gap-by-masking condition interaction were also included. The significance of a given effect (random or fixed) was tested by likelihood ratio comparison with a null model where the effect was excluded.

### Results

The quiet threshold for the 5-ms, 2-kHz sinusoidal signal, used to set the signal level for the TMC measurements (10 dB SL), was 23.5 ± 0.90 (mean ± standard error) dB SPL on average. The average tympanometric threshold was 85 ± 3.1 dB SPL, and the lowest individual threshold was 70 dB SPL. Tympanometric measurements may overestimate the MEM reflex threshold by up to 15 dB (Goodman and Keefe 2006). Therefore, the elicitor, an equally exciting noise (see “Methods” section), was set to a level of 40 dB SPL per ERB_N_, corresponding to 54 dB SPL overall, just below the lowest individual MEM reflex threshold less 15 dB.

**FIG. 3:**
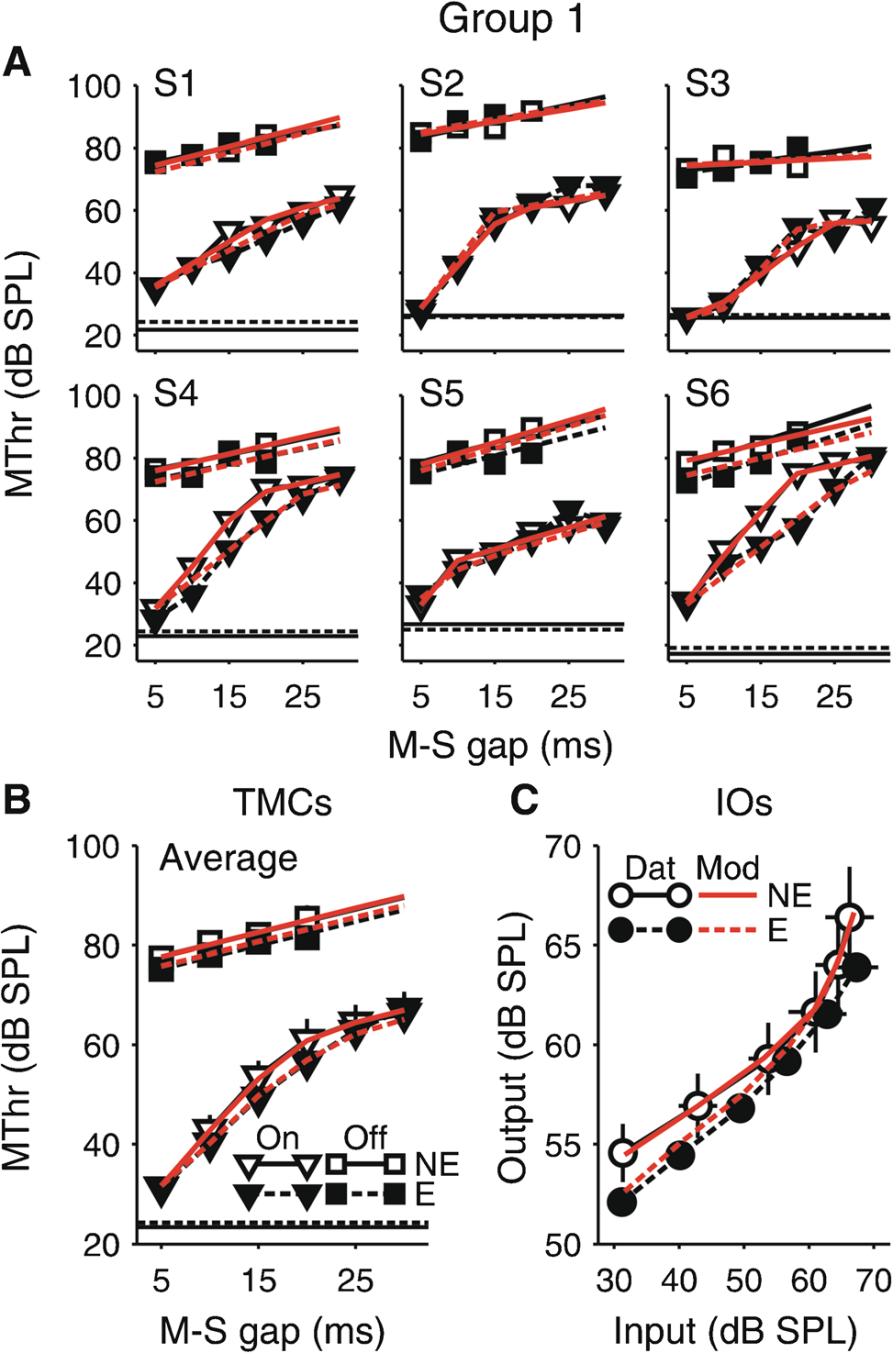
TMC results for the first group of subjects (S1–S6), who were tested in both the on- and off-frequency conditions with and without the elicitor. **A**, **B** Individual and average TMCs as a function of masker-signal gap (M-S gap), and **C** average inferred IO function. The data are shown in *black* (Dat). The *red lines* (Mod) show model fits explained in a later section (“Cochlear IO Function Model” section). The on- and off-frequency masking thresholds (On, Off) are shown by *triangles and squares*, respectively. The without-elicitor TMCs (*NE*) are shown by *open symbols* and *solid lines* and the with-elicitor TMCs (*E*) by *closed symbols* and *dashed lines* (legends in **B** and **C**). The IO functions in panel **C** were constructed by plotting the off-frequency masking threshold for each masker-signal gap against the corresponding on-frequency threshold and correcting for the passive difference between the on- and off-frequency masker responses at the signal place (*P*; derived from the cochlear IO function model fits). The *error bars* in panels **B** and **C** show the standard error of the mean (SEM).

**FIG. 4:**
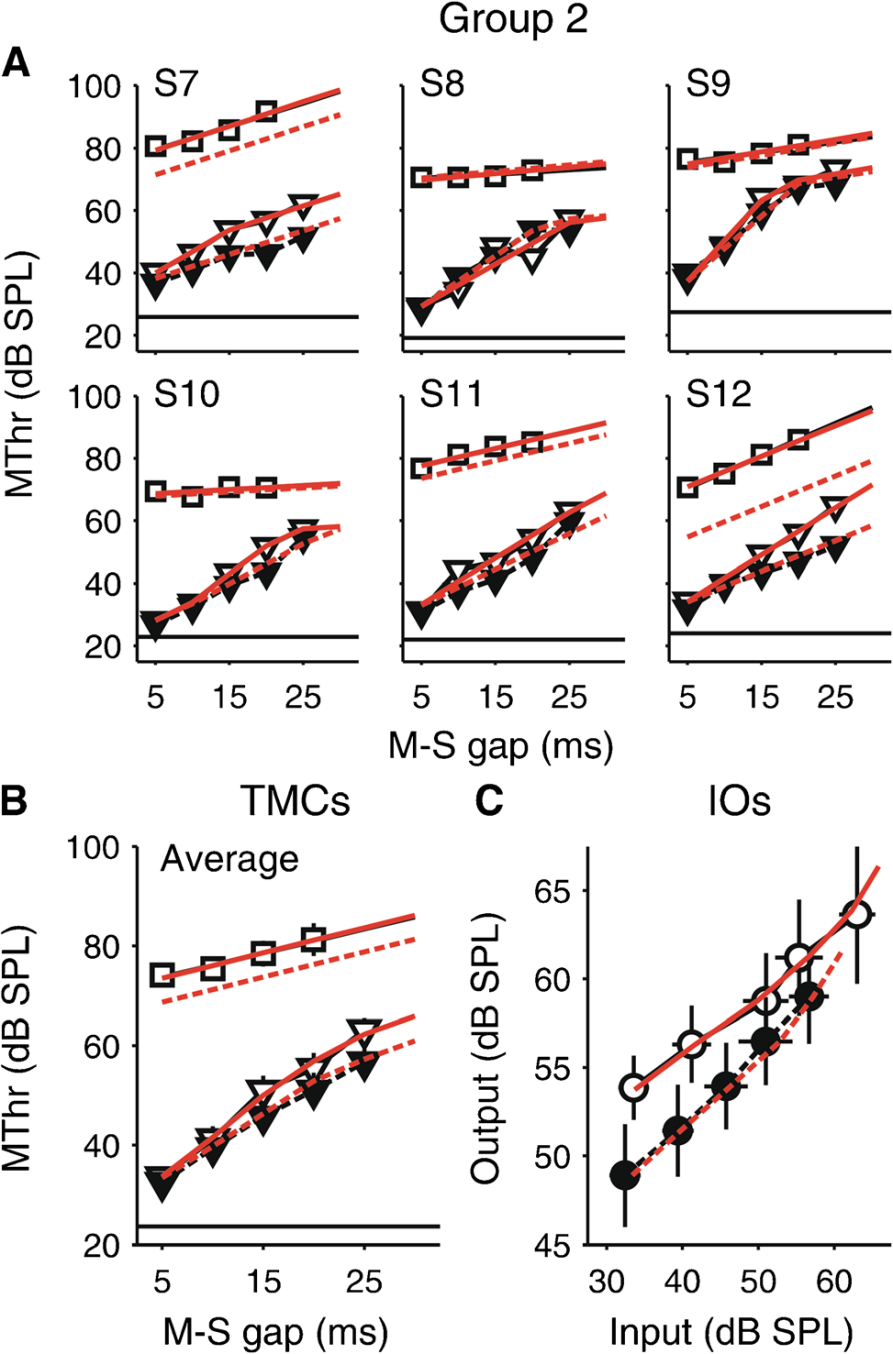
TMC results for the second group of subjects (S7–S12), plotted in the same way as the results for the first group (Fig. 3). In the second group, the off-frequency TMC was measured only without the elicitor. The with-elicitor IO function in panel **C** was constructed using the predicted off-frequency TMCs based on the cochlear IO function model fits (linear *red dashed lines* in panels **A** and **B**).

The subjects were split into two groups. The first group (S1–S6) measured both the on- and off-frequency TMCs with and without the elicitor, whereas the second group (S7–S12) measured the off-frequency TMC only without the elicitor. The individual and averaged data from the two groups are shown in Figures 3 and 4.

#### Without-Elicitor TMCs

As expected, the TMCs without the elicitor (open symbols and solid lines in Figs. 3 and 4, panels A and B) showed a clear effect of masking condition: not only were the on-frequency masking thresholds generally lower than the off-frequency thresholds (tested with a MLM of the without-elicitor thresholds from all subjects (see “Methods” section); main effect of masking condition: *χ* ^2^(1) = 32.575, *p* < 0.001), but they also increased more steeply with increasing masker-signal gap (masking condition-by-linear gap interaction: *χ* ^2^(1) = 29.249, *p* < 0.001) and showed a greater degree of non-linearity (masking condition-by-quadratic gap interaction: *χ* ^2^(1) = 9.967, *p* = 0.0016). The off-frequency thresholds increased linearly with increasing masker-signal gap at an average rate, *μ*, of 0.5 dB/ms (separate MLM of the off-frequency thresholds; main effect of linear gap: *χ* ^2^(1) = 15.573, *p* < 0.001; main effect of quadratic gap: *χ* ^2^(1) = 1.121, *p* = 0.29). In contrast, the on-frequency thresholds increased nonlinearly with increasing masker-signal gap (separate MLM of the on-frequency thresholds; main effect of quadratic gap: *χ* ^2^(1) = 29.626, *p* < 0.001). The slope of the on-frequency TMC was steeper than that of the off-frequency TMC at short and intermediate masker-signal gaps, but the slopes converged towards longer gaps. The fact that the on-frequency TMC did not show a shallower slope at the shortest gaps measured indicates that we failed to sample the initial linear part of the on-frequency masker IO function. This is because we used a shorter masker duration to avoid eliciting the MOC reflex, and shorter maskers are less effective, and thus associated with higher masking thresholds.

**FIG. 5:**
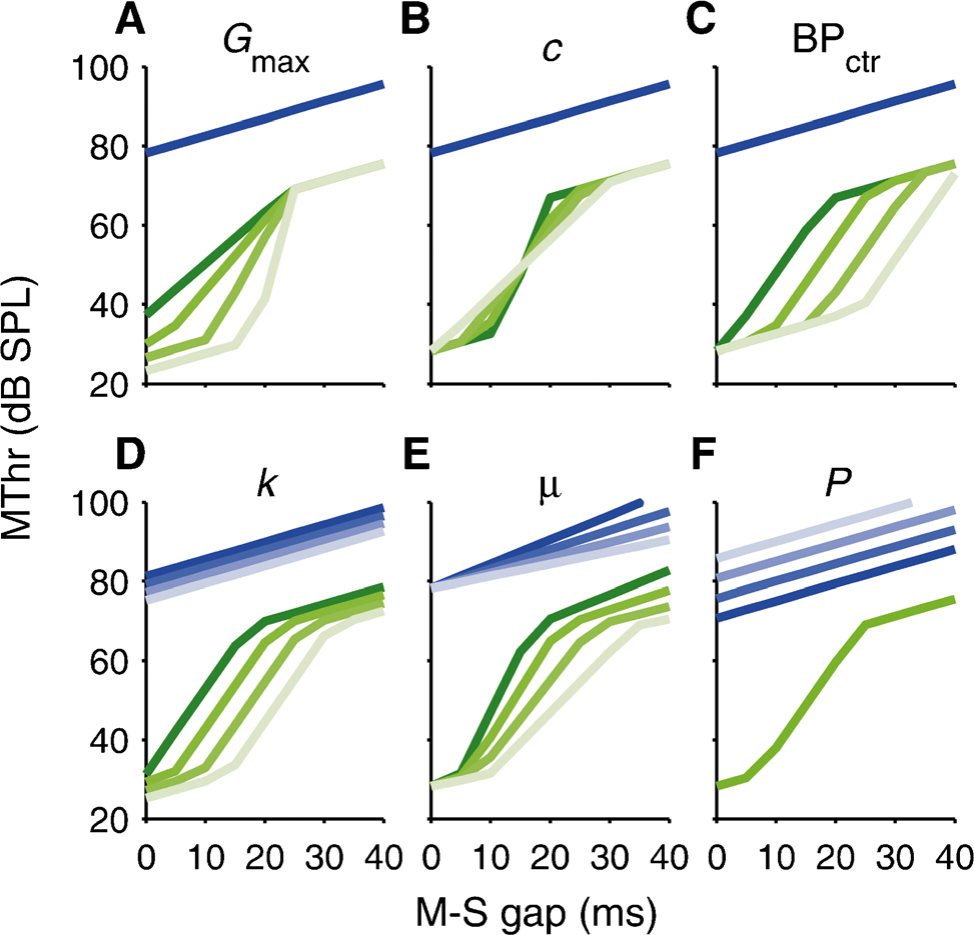
Effect on TMCs of variation in each model parameter (maximum cochlear gain, *G*_max_, compression exponent, *c*, center of compressive range, BP_ctr_, threshold signal-to-masker ratio, *k*, masker effect decay rate, *μ*, and passive attenuation, *P*). For each panel, each parameter was varied separately. When varying *G*_max_ **(A)**, the compressive range (defined by the break points, BP_1_ and BP_2_) was kept fixed, and so the compression exponent, *c*, had to co-vary. When varying *c* (**B**), *G*_max_ was kept fixed, and so the compressive range had to co-vary.

The TMCs showed a considerable degree of inter-individual variability (Figs. 3A and 4A). A MLM analysis showed significant by-subject random effects of (i) the linear masker-signal gap (*χ* ^2^(2) = 13.066, *p* = 0.0015), (ii) the masking condition (*χ* ^2^(3) = 34.76, *p* < 0.001), and (iii) the linear gap-by-masking condition interaction (*χ* ^2^(4) = 13.058, *p* = 0.011). These effects show that subjects differed in terms of (i) the average TMC slope across masking conditions, (ii) the relative positions along the ordinate of the on- and off-frequency TMCs, and (iii) the relative slopes of the on- and off-frequency TMCs. In the upcoming modeling section (“Cochlear IO Function Model” section), we present an analysis suggesting that these effects are caused by different physiological factors: (i) the average TMC slope is determined by the decay rate, *μ*, of the masker effect over time (see Fig. 5E); (ii) the relative positions along the ordinate of the on- and off-frequency TMCs are determined by the passive attenuation, *P*, of the off-frequency masker tail response (Fig. 5F); and (iii) the relative slopes of the on- and off-frequency TMCs are determined by the cochlear gain, *G*_max_, and compression exponent, *c*, of the on-frequency masker tip response (Fig. 5A, B).

#### Effect of the Elicitor

Both the on- and off-frequency masking thresholds were generally lower with than without the elicitor (MLMs of the on-frequency masking thresholds from all 12 subjects and the off-frequency thresholds from S1–S6; main effects of elicitor condition: *χ* ^2^(1) = 6.949, *p* = 0.0083, and *χ* ^2^(1) = 8.052, *p* = 0.0045, respectively; compare closed and open symbols in Figs. 3 and 4, panels A and B). The data from the first group (S1–S6; Fig. 3A, B) show that the elicitor caused the off-frequency masking thresholds to decrease about equally across all masker-signal gaps (elicitor-by-linear and elicitor-by-quadratic gap interactions: both *χ* ^2^(1) ≤ 0.017, *p* ≥ 0.896). In the on-frequency condition, the elicitor effect varied with the masker-signal gap: the effect was absent at the shortest gap, increased towards intermediate gaps, and then decreased again towards longer gaps. As a result, the on-frequency TMCs became shallower (elicitor-by-linear gap interaction: *χ* ^2^(1) = 11.429, *p* < 0.001) and less non-linear (elicitor-by-quadratic gap interaction: *χ* ^2^(1) = 10.751, *p* = 0.001).

As a result, the inferred IO functions with and without the elicitor (Figs. 3C and 4C) showed the largest difference at low input levels, and a decreasing difference towards higher levels (for the second group (Fig. 4C), the with-elicitor IO function was constructed using a predicted without-elicitor off-frequency TMC based on model fits described in the next section). Thus, the IO functions with the elicitor grew less compressively than those without the elicitor. This pattern of results is consistent with the idea that the elicitor caused a reduction in cochlear gain.

Like the without-elicitor TMCs, the elicitor effect showed considerable inter-individual variability (MLM of the on-frequency masking thresholds, which were measured with and without the elicitor in all 12 subjects; by-subject random effects of elicitor condition: *χ* ^2^(3) = 25.547, *p* < 0.001), with some subjects showing little or no effect (e.g., S2 (Fig. 3A) and S8 (Fig. 4A)), and some showing particularly large effects (e.g., S6 (Fig. 3A) and S12 (Fig. 4A)).

In the first group (S1–S6; Fig. 3), the signal detection threshold in quiet was measured with and without the elicitor (horizontal lines in Fig. 3A, B). On average, the threshold was 0.78 ± 0.622 dB higher with than without the elicitor. The difference was statistically non-significant (paired *t* test; *t*(6) = 2.38, *p* = 0.817).

#### Cochlear IO Function Model

In order to derive the amount of elicitor-induced reduction in cochlear gain, we fitted the on- and off-frequency TMCs with a generic model of the cochlear tip and tail response IO functions. Fitting the TMCs, rather than the inferred IO function as in previous studies (e.g., Plack et al. 2004), circumvents the problem that individual inferred IO functions can be non-monotonic due to non-monotonicity in the often sparse and noisy off-frequency TMCs (e.g., S3 in Fig. 3A, S10 in Fig. 4A). The tip response IO function (applicable to the signal and on-frequency masker) was assumed to be equal to a piecewise linear function, *f*_*a*_, of the input sound level, *L* (Yasin and Plack 2003). *f*_*a*_ was equal to *L* plus a level-dependent gain, *G*(*L*). In units of intensity, this becomes ${f}_a(L)={10}^{\left(L+G(L)\right)/10}$. *G*(*L*) was constant and maximal up to a first break point, BP_1_: *G*(*L* ≤ BP_1_) = *G*_max_, and then decreased linearly to zero, at a rate of 1 − *c*, where *c* is the compression exponent, between BP_1_ and a second break point, BP_2_,: *G*(BP_1_ ≤ *L* ≤ BP_2_) = (*c* − 1)(*L* − BP_1_) + *G*_max_. Above BP_2_, *G*(*L*) was assumed to remain zero: *G*(*L* ≥ BP_2_) = 0. The tail response IO function (applicable to the off-frequency masker), *f*_*p*_, was assumed to be equal to the input sound level, *L*, minus a constant, *P*, representing any passive attenuation of the tail, compared to the tip, response: *f*_*p*_(*L*) = 10^(*L* − *P*)/10^ (note that *P* was not constrained to be positive). The masker effect (denoted as *E*) was assumed to decay exponentially, at a rate *μ*, with increasing masker-signal gap, *t*: *E*(*t*) = *E*(*t* = 0) ⋅ *e* ^− *μ* ⋅ *t*^. The masking threshold was assumed to correspond to a constant ratio, *k*, between the signal and masker responses. Using these assumptions, the on- and off-frequency masking thresholds, *MThr*_on_ and *MThr*_off_, for each masker-signal gap, *t*, were predicted as *MThr*_on_(*t*) = *f*_*a*_^− 1^(*f*_*a*_(*L*_*s*_)/(*k* ⋅ *e* ^− *μ* ⋅ *t*^)) and *MThr*_off_(*t*) = *f*_*p*_^− 1^(*f*_*a*_(*L*_*s*_)/(*k* ⋅ *e* ^− *μ* ⋅ *t*^)), where *f*_*a*_^− 1^ and *f*_*p*_^− 1^ are the inverse of the functions *f*_*a*_ and *f*_*p*_, respectively, and *L*_*s*_ is the signal pressure level.

Figure 5 shows that variation in each model parameter creates a distinct pattern of variation in the predicted TMCs. Variation in *G*_max_ and *c* causes variation in the slope of the non-linear section of the on-frequency TMC, and variation in the position of the compressive range, BP_ctr_ = (BP_1_ + BP_2_)/2, causes this section to shift along the abscissa (Fig. 5A–C). All three parameters leave the off-frequency TMC unchanged. In contrast, variation in *k* and *μ* causes equal variation in both TMCs, in the position along the abscissa and the TMC slope, respectively (Fig. 5D, E), and variation in *P* only affects the off-frequency TMC, shifting its position along the ordinate (Fig. 5F).

To fit the without-elicitor thresholds, the sum of the squared differences between all observed and predicted thresholds (on- and off-frequency) was minimized by varying the maximum gain, *G*_max_, the compression exponent, *c*, the center of the compressive range, BP_ctr_, the signal-to-masker ratio, *k*, the masker decay rate, *μ*, and the passive attenuation, *P*. The fitting was conducted separately for each subject using *lsqnonlin* in MATLAB. To fit the with-elicitor TMCs, the elicitor was assumed to reduce *G*_max_ by *ΔG*, with *ΔG* a free parameter. Note that, in order not to bias the model outcome, *ΔG* was not constrained to be positive (i.e., the with-elicitor gain was not constrained to be smaller than the without-elicitor gain). All other model parameters (i.e., the first and second break points, BP_1_ and BP_2_, the passive off-frequency masker attenuation, *P*, the signal-to-masker ratio, *k*, and the decay rate of the masker effect, *μ*) were carried over from the without-elicitor fits. The fact that the break points were fixed meant that the compression exponent, *c*, changed according to $\tilde{c}=1-\left({G}_{\max }-\varDelta G\right)/\left({\mathrm{BP}}_2-{\mathrm{BP}}_1\right)$. In the first group of subjects (S1–S6; Fig. 3), the fitting of *ΔG* was based on both the on- and off-frequency thresholds, whereas in the second group (S7–S12), the fitting was based only on the on-frequency thresholds. To test whether this influenced the fitting results, we repeated the fitting in the first group with the off-frequency thresholds omitted. The new fits were practically identical to the original ones (data not shown), and the best-fitting parameter (*ΔG*) was statistically indistinguishable (Wilcoxon signed-rank; *p* = 1). *ΔG* was also not significantly different between the two subject groups (Wilcoxon rank sum; *p* = 0.589). This confirms that a reliable estimate of the elicitor-induced gain reduction can be achieved with the on-frequency TMC alone.

**FIG. 6:**
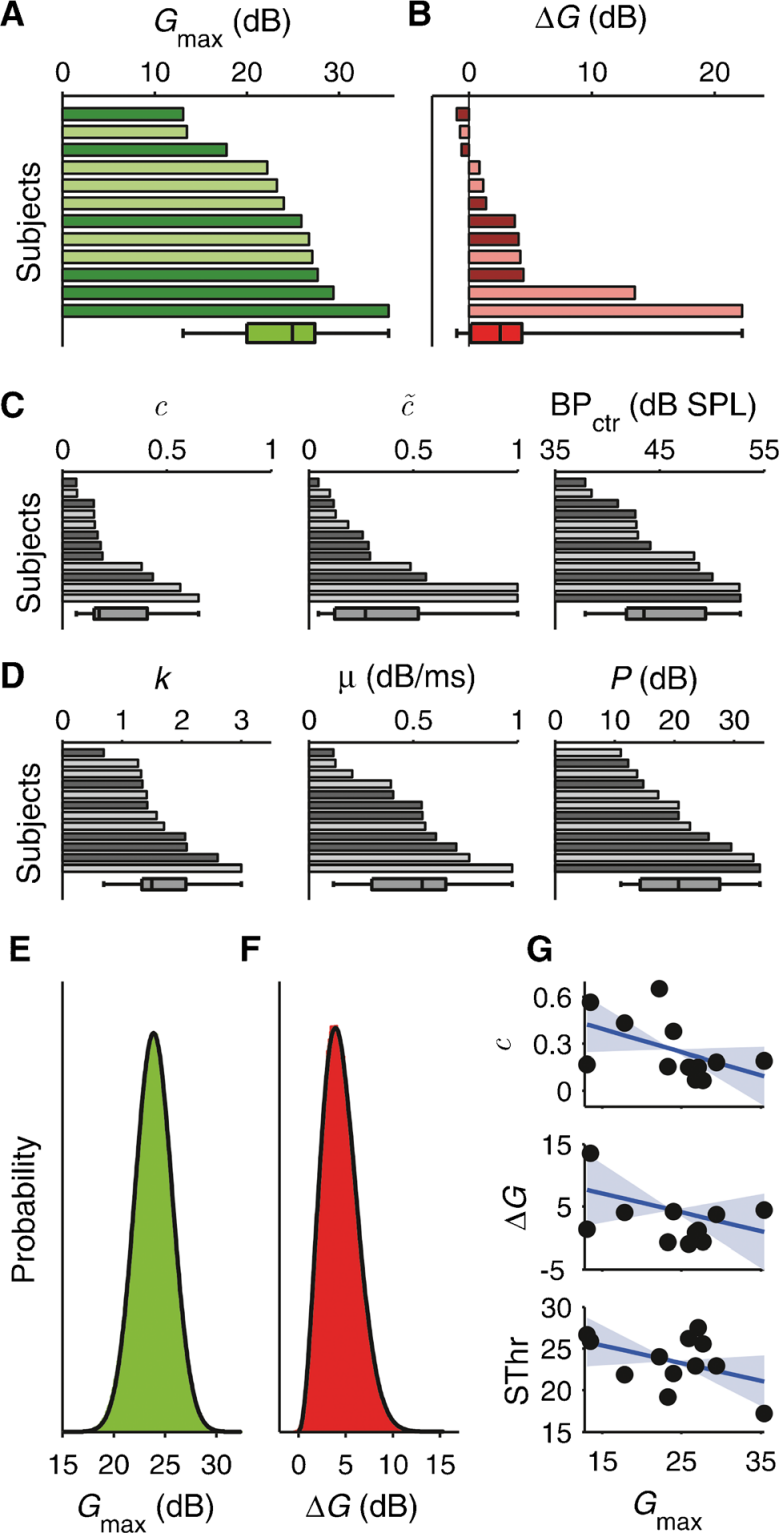
**A**–**D** Individual best-fitting model parameters, *G*_max_ (maximum without-elicitor gain; **A**), *ΔG* (elicitor-induced gain reduction; **B**), *c* (without-elicitor compression exponent; **C**, *left*), $\tilde{c}$ (with-elicitor compression exponent; **C**, middle), BP_ctr_ (center of compressive range; **C**, *right*), *k* (threshold signal-to-masker ratio; **D**, *left*), *μ* (decay rate of masker effect; **D**, *center*), and *P* (passive attenuation; **D**, *right*). The parameters were sorted for size (independently in each panel). The *bar and whiskers* at the bottom of each panel show the median, 25th and 75th percentile, and minimum and maximum parameter values. The *darker-shaded bars* are the results of the first group of subjects (S1–S6; see Fig. 3), and the *lighter-shaded bars* are the results for the second group (S7–S12; see Fig. 4) **(E, F).** Bootstrap distributions of *G*_max_ and *ΔG* (*green and red*; based on all possible $$ \left(\begin{array}{c}\hfill 2N-1\hfill \\ {}\hfill N\hfill \end{array}\right) $$ bootstrap resamples, where *N* = 12 is the number of subjects). The *black lines* show the best-fitting probability density functions. **G** Across-subject relationships between *G*_max_ and *c* (*top*), *G*_max_ and *ΔG* (*middle*), and *G*_max_ and the signal quiet threshold (SThr) in dB SPL. The *blue solid lines* are the regression lines. The *light-blue highlight* shows the bootstrap confidence intervals of the regression slopes (again, based on all possible $$ \left(\begin{array}{c}\hfill 2N-1\hfill \\ {}\hfill N\hfill \end{array}\right) $$ resamples).

The red lines in Figures 3 and 4 show that the model produced an excellent fit to both the with- and without-elicitor data. The root-mean-square deviation (RMSD) between the average observed and predicted thresholds was 1.67 dB. The individual RMSDs ranged from 1.05 to 2.67 dB. The individual and median model parameters are shown in Figure 6 and listed in Table 1.

**TABLE 1:** Best-fitting model parameters for each subject

	*G*_max_	*c*	BP_ctr_	*k*	*μ*	*P*	*ΔG*	$\tilde{c}$
S1	17.80	0.43	42.68	1.34	0.61	25.69	4.04	0.56
S2	25.92	0.15	44.11	2.06	0.40	29.48	−0.98	0.12
S3	27.69	0.07	41.00	1.42	0.12	20.70	−0.60	0.05
S4	29.41	0.18	50.07	2.08	0.54	14.74	3.74	0.29
S5	13.08	0.17	37.88	2.60	0.71	34.33	1.39	0.26
S6	35.39	0.19	52.74	0.69	0.54	12.26	4.43	0.29
S7	13.50	0.56	38.50	1.42	0.77	33.19	13.50	1.00
S8	23.28	0.15	42.93	1.27	0.21	17.29	−0.71	0.13
S9	27.11	0.15	52.65	1.58	0.39	11.01	1.18	0.19
S10	26.75	0.07	42.79	1.32	0.13	13.75	0.87	0.10
S11	23.99	0.38	48.77	1.70	0.56	22.59	4.18	0.49
S12	22.21	0.65	48.31	3.00	0.97	20.66	22.21	1.00
Median	23.64	0.18	43.52	1.50	0.54	20.68	2.57	0.27

*Abbreviations*: *G*_*max*_ maximum cochlear gain without elicitor (dB), *c* without-elicitor compression exponent, BP_ctr_ center of compressive range (dB SPL), *k* signal-to-masker ratio at threshold, *μ* masker effect decay rate (dB/ms), *P* passive attenuation of off-frequency masker response (dB), *ΔG* elicitor-induced gain reduction (dB), $\tilde{c}$ with-elicitor compression exponent

The best-fitting model parameters reflected the inter-individual variability in the without-elicitor TMCs and elicitor effect (Fig. 6A–E; see also Table 1). In individual subjects, the without-elicitor cochlear gain, *G*_max_, ranged from 13.1 to 35.4 dB (Fig. 6A), and the without-elicitor compression exponent, *c*, ranged from 0.07 to 0.65 (Fig. 6C, left). The elicitor-induced gain reduction, *ΔG*, ranged from −1 to 22.2 dB (−3.8 to 100 % of the without-elicitor gain; Fig. 6B), and the with-elicitor compression exponent, $\tilde{c}$, from 0.05 to 1.0 (Fig. 6C, middle). Despite being permitted to become negative, *ΔG* was generally either very close to, or larger than zero, suggesting that the population distribution of *ΔG* is positively skewed. This was confirmed by the bootstrap distribution of the average *ΔG* across subjects (Fig. 6F; the bootstrap distribution represents the expected distribution of average *ΔG* values if the experiment were repeated many times, with different groups of subjects). While the bootstrap distribution of the average *G*_max_ (Fig. 6E) was best fitted by a symmetrical Gaussian probability density function (*pdf*) with mean and standard deviation *μ* = 23.9 dB and *σ =* 1.80 dB, the bootstrap distribution of the average *ΔG* was best fitted by a positively skewed Nakagami *pdf *with shape and spread parameters *m* = 1.52 and *Ω* = 23.44 dB^2^ (Kolar et al. 2004). This distribution has an expected value (mean) of 4.4 dB, corresponding to 18.4 % of the mean *G*_max_ (23.9 dB). As a result of the gain reduction, the median compression exponent increased from 0.18 to 0.27 (see Table 1 and Fig. 6).

The average without-elicitor gain estimate (23.9 dB) is smaller (by ∼20 dB) than most of the previous psychophysical gain estimates (reviewed in Yasin et al. 2013). This is because the current modeling allowed for part of the difference between the on- and off-frequency masking thresholds to be explained by passive filtering (parameter *P*). Discounting *P* increased the average without-elicitor gain estimate to 43.9 dB, which is more similar to the previous estimates. However, this decreased the goodness of fit (RMSD = 0.39 dB for the original model, compared to 1.93 dB for the model with *P* = 0), albeit non-significantly (*F*(1,7) = 0.22, *p* = 0.652).

In the without-elicitor conditions, there was a tendency, albeit marginal (Spearman’s rank order correlation, *R*_S_ = −0.46, *p* = 0.067), for the compression exponent, *c*, to decrease with increasing cochlear gain, *G*_max_ (average rate −0.15 per 10 dB; Fig. 6G, top), as might be expected (e.g., Baker and Rosen 2002). If MOC-induced reduction in cochlear gain were multiplicative in decibel units, the amount of gain reduction (*ΔG*) would be expected to increase with increasing *G*_max_. In fact, however, *ΔG* changed little with increasing *G*_max_ (average rate −0.30 dB per dB; Fig. 6G middle; *R*_S_ = −0.22, *p* = 0.765), suggesting that MOC-induced gain reduction may be multiplicative in linear units. Finally, the signal quiet threshold (SThr; Fig. 6G, bottom) tended to decrease with increasing *G*_max_, as would be expected (Plack et al. 2004), but the rate of decrease was only −0.21 dB per dB on average. Based on this rate, the elicitor would be predicted to increase the signal quiet threshold by 0.95 dB (assuming a gain reduction, *ΔG*, of 4.4 dB) close to the actually measured increase of 0.78 dB. The correlation between the signal quiet threshold and *G*_max_ was non-significant (*R*_S_ = −0.23, *p* = 0.238).

#### Separate Versus Interleaved Sessions

**FIG. 7:**
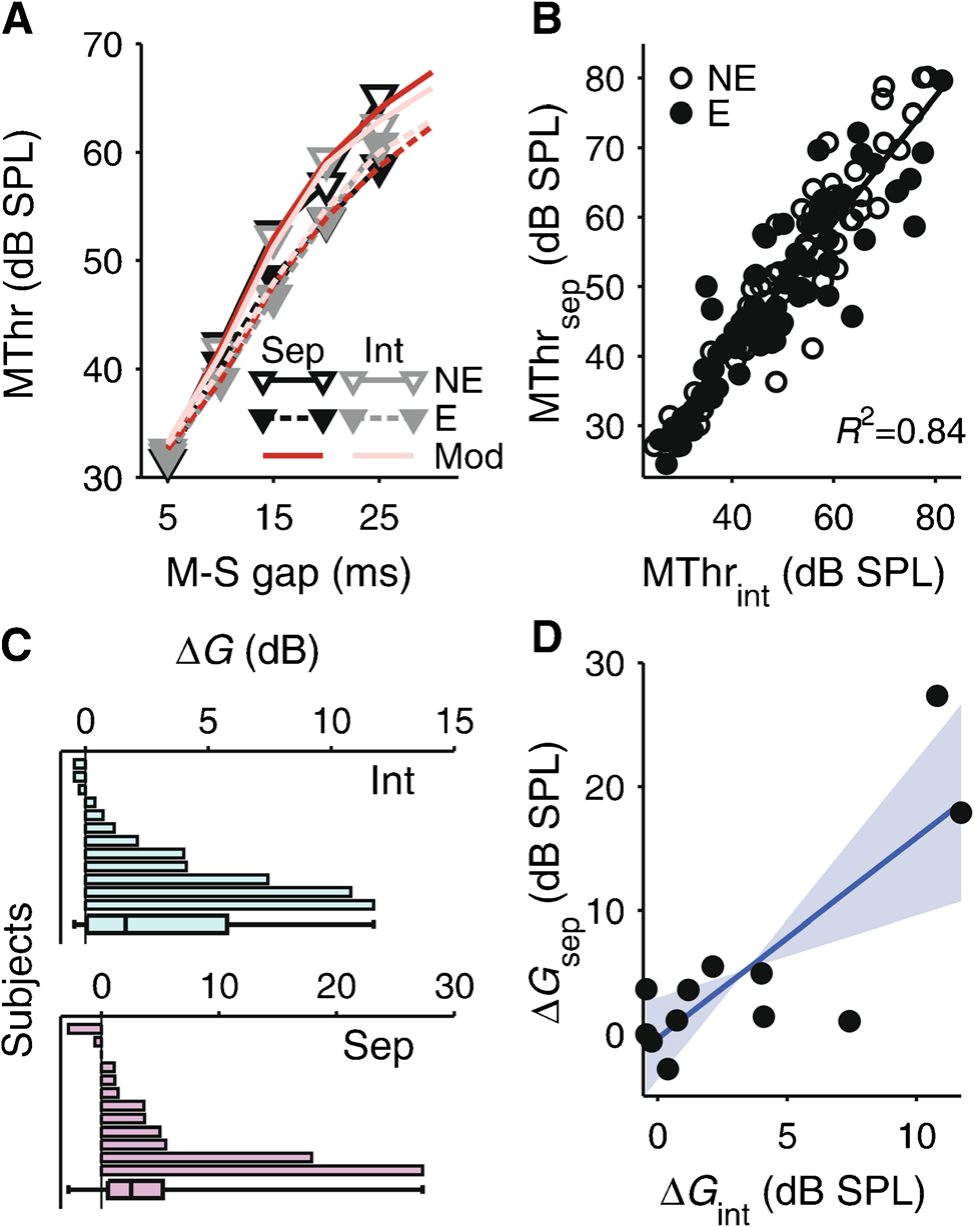
**A** Comparison between on-frequency TMCs measured in the separate and interleaved sessions. The TMCs from the separate sessions (Sep) are shown in *black* and those from the interleaved sessions (Int) in *gray*. The corresponding model fits (Mod) are shown by *darker- and lighter-red lines*. The without-elicitor TMCs (*NE*) are shown by *open symbols and solid lines* and the with-elicitor TMCs (E) by *closed symbols and dashed lines* (legend). **B** Relationship between the masking thresholds from the interleaved and separate sessions (MThr_int_, MThr_sep_). The *open circles* show the without-elicitor and the *filled circles* the with-elicitor, thresholds (legend). The *solid line* is the regression line. *R* ^2^ is the squared Pearson correlation coefficient. **C** Individual elicitor-induced gain reductions, *ΔG*, for the interleaved (*top*) and separate (*bottom*) sessions, sorted by size (independently, as before). The *bar and whiskers* at the bottom of each panel show the median, percentiles, and absolute range as in Figure 6. **D** Across-subject relationship between *ΔG* for the interleaved and separate sessions (denoted *ΔG*_int_ and *ΔG*_sep_). The *blue solid line* is the regression line, and the *light-blue highlight* shows the bootstrap confidence interval of the regression slope as in Figure 6.

The on-frequency TMCs with and without the elicitor were measured either in separate or interleaved sessions. Figure 7 shows that the results from the two session types were highly similar (compare black and gray symbols in panel A). The masking thresholds from one session type accounted for 84 % of the variance in the masking thresholds from the other session type (Fig. 7B). Separate MLM analyses of the with- and without-elicitor TMCs showed no significant main effects of session type (both *χ* ^2^(1) ≤ 0.455, *p* ≥ 0.50) or interactions with linear (both *χ* ^2^(1) ≤ 1.643, *p* ≥ 0.20) or quadratic masker-signal gap (both *χ* ^2^(1) ≤ 2.702, *p* ≥ 0.1002). The data from the two session types were fitted separately with the cochlear IO function model. The best fitting model parameters were not significantly different from each other (Wilcoxon signed-rank tests; all *p* ≥ 0.291). For the separate sessions, the elicitor-induced gain reduction, *ΔG*, averaged 5.3 dB (−2.83–27.31 dB range), and for the interleaved sessions, *ΔG*, averaged 3.4 dB (−0.44–11.71 dB range; Fig. 7C). *ΔG* showed a significant across-subject correlation between the two session types (*R*_S_ = 0.62, *p* = 0.017; Fig. 7D).

## **EXPERIMENT 2: CONTRALATERAL SUPPRESSION OF OTOACOUSTIC EMISSIONS**

In this experiment, we measured the effect of the contralateral MOC elicitor used in experiment 1 on OAEs evoked by a click stimulus. Click-evoked OAEs (CEOAEs) contain energy within a frequency region between ∼0.5 and 4 kHz (e.g., Bonfils et al. 1988), which overlaps the 2-kHz signal frequency used in experiment 1. The probe was presented at a level of 60 or 70 dB peak equivalent (pe) SPL. Using two probe levels makes it possible to measure the compressiveness of the growth in OAE amplitude with increasing probe level (Veuillet et al. 1991). Activation of the MOC reflex reduces the amplitude of OAEs (referred to as “amplitude suppression”; Guinan 2010) and has also been shown to reduce the compressiveness of the OAE growth with probe level (referred to as “IO suppression”; Veuillet et al. 1996).

Experiment 2 was conducted after experiment 1, using the same group of subjects. Again, the with- and without-elicitor conditions were measured either in different sessions, separated by longer breaks, or interleaved within a single session. In this experiment, all sessions were conducted on the same day.

### Methods

#### OAE Measurements

CEOAEs were recorded with an in-house system (MLS 2001) consisting of a digital signal-processing board controlled by a custom-written software (Visual Basic). The clicks had a 100-μs duration and were generated at a 30-kHz sampling rate. They were presented at a rate of 20/s using a general purpose OAE transducer (Otodynamics, Hatfield, UK). The OAEs were recorded using the transducer microphone and digitized with a 30-kHz sampling rate and 18-bit amplitude resolution. They were averaged online over 2000 trials. Two such averages (referred to as “replicates”) were recorded for each click level (60 and 70 dB pe SPL) and elicitor condition (present/absent). Trials were rejected if the response amplitude exceeded 5 mPa within the period from 6 to 16 ms after the click. Each replicate took ∼2 min to acquire, similar to the adaptive tracks in experiment 1. The elicitor was presented continuously throughout this period. It was filtered in the same way and presented at the same level and through the same headphones (Sennheiser HD 600), as in experiment 1. Within a session, replicates were measured contiguously. Different sessions were separated by breaks of at least 5 min. The OAE measurements were performed in the same sound-attenuating booth as experiment 1. Subjects watched a silent subtitled movie of their own choice to stay alert.

#### OAE Data Analysis

Offline analysis of OAEs was performed in MATLAB. First, the OAEs were filtered between 250 and 6 kHz by applying a second-order Butterworth filter in both forward and reverse time direction to create zero phase delay. To minimize the stimulus artifact, the OAEs were windowed between 6 and 16 ms after the click. The window edges were rounded with 2-ms quarter-sine and -cosine functions. The CEOAE amplitude for each condition was taken as the integral of the co-spectrum (real part of cross-spectrum) between the respective replicates (Marshall and Heller 1996). A CEOAE was accepted as valid only if the correlation between the two replicates (referred to as “reproducibility”) was greater than 0.7. The reproducibility for all included CEOAEs averaged to 0.95 ± 0.007. CEOAE suppression by the contralateral elicitor was quantified using the normalized index, ΔCEOAEn, which is the elicitor-induced change in CEOAE amplitude (in linear units) as a proportion of the without-elicitor amplitude in percent (Mishra and Lutman 2014).

Like the masking thresholds, the CEOAE amplitudes were analyzed with MLMs, implemented in *R*. The models included fixed factor effects of click level (60/70 dB pe SPL), session type (separate/interleaved), and elicitor condition (with/without), as well as random by-subject slopes for click level, elicitor condition, and session type (the latter was omitted when the data for each session type were analyzed separately).

### Results

**FIG. 8:**
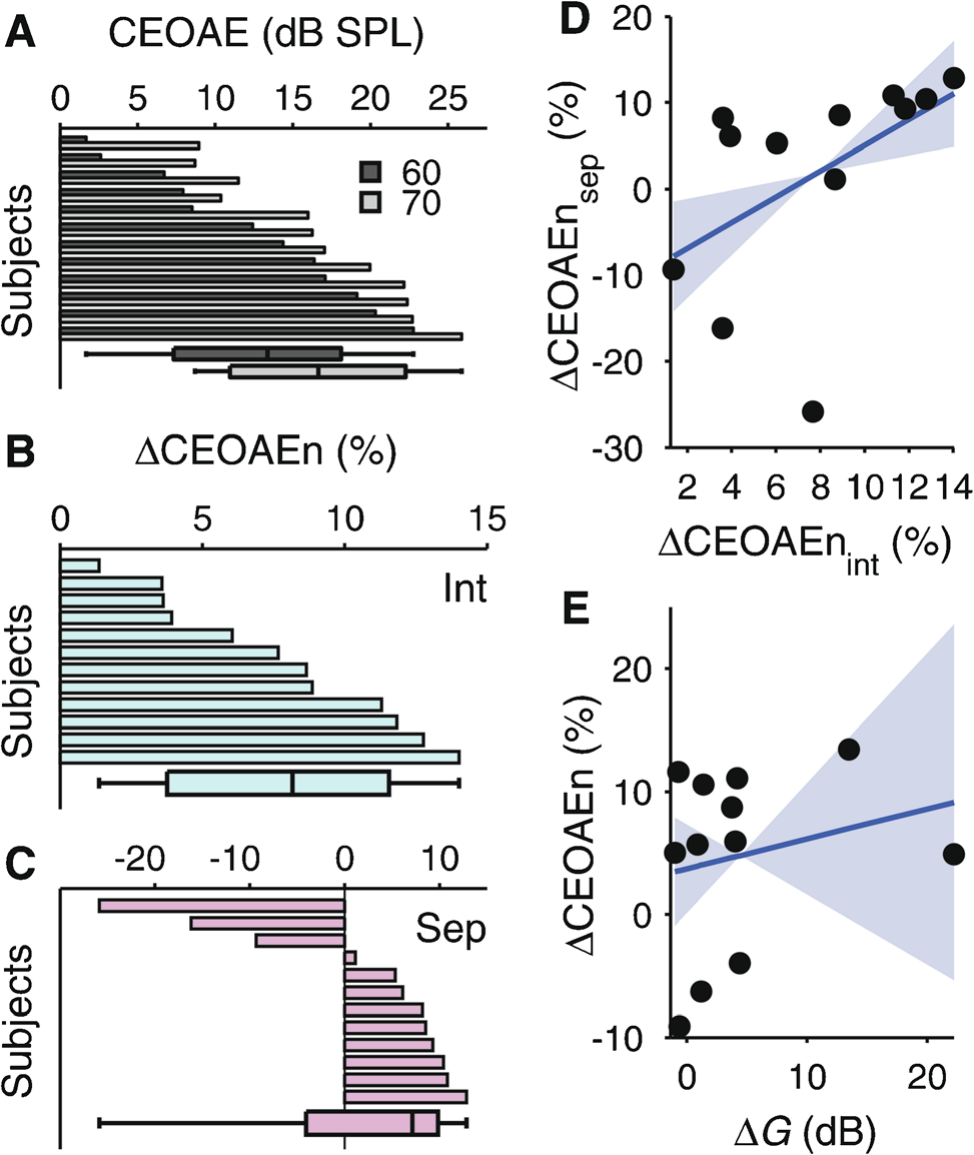
**A** Individual without-elicitor CEOAE amplitudes for the 60- and 70-dB pe SPL click levels (*dark- and light-gray bars*), sorted in order of the size of the CEOAE amplitudes for the 60-dB pe SPL clicks. **B**, **C** Individual normalized CEOAE suppression indices, ΔCEOAEn (Mishra and Lutman 2014; negative values of ΔCEOAEn denote elicitor-induced CEOAE enhancement), for the interleaved **(B)** and separate **(C)** sessions, sorted for size (independently). The *bars and whiskers* at the bottom of each panel show the respective medians, percentiles, and absolute ranges as in the previous figures. **D** Across-subject relationship between ΔCEOAEn for the interleaved and separate sessions. **E** Across-subject relationship between the elicitor-induced gain reductions, *ΔG*, estimated from the TMCs, and ΔCEOAEn, averaged across sessions. The *blue, solid lines* show the regressions lines and the *light-blue highlight* the bootstrap confidence intervals of the regression slopes.

The CEOAE amplitudes were generally larger for the 70- than 60-dB pe SPL clicks (MLM of CEOAE amplitudes in dB SPL; main effect of click level *χ* ^2^(1) = 23.873, *p* < 0.001; see Fig. 8A), and they were also generally smaller for the with- than the without-elicitor conditions (amplitude suppression; main effect of elicitor condition *χ* ^2^(1) = 5.476, *p* = 0.019). Somewhat surprisingly, the elicitor effect did not depend on the click level (no IO suppression; elicitor condition-by-click level interaction *χ* ^2^(1) = 0.018, *p* = 0.894). This was true irrespective of whether the with- and without-elicitor conditions were measured in separate or interleaved sessions (three-way interaction between elicitor condition, click level and session type *χ* ^2^(1) = 0.019, *p* = 0.889). The elicitor effect itself, however, did depend on the order in which the with- and without-elicitor conditions were measured (elicitor condition-by-session type interaction *χ* ^2^(1) = 9.473, *p* = 0.0021). The elicitor caused significant CEOAE amplitude suppression in the interleaved session (Fig. 8B), but not in the separate sessions (Fig. 8C; separate MLM for each session type; elicitor condition main effect for interleaved session *χ* ^2^(1) = 18.435, *p* < 0.001; but for separate sessions *χ* ^2^(1) = 0.566, *p* = 0.452). In the interleaved session, the amplitude suppression (expressed as normalized suppression index, ΔCEOAEn; Mishra and Lutman 2014) averaged 7.80 % and ranged from 1.36 to 14.02 % across subjects. In the separate sessions, the suppression averaged only 1.77 % and ranged from −25.88 to 12.83 % (a negative suppression index means elicitor-induced CEOAE enhancement).

The session type effect on ΔCEOAEn contrasts with the absence of any session type effect on *ΔG* in experiment 1. However, like *ΔG*, ΔCEOAEn showed a significant across-subject correlation between the two session types (Fig. 8D; *R*_S_ = 0.776, *p* = 0.0023). Figure 8E shows that there was a tendency for ΔCEOAEn to increase with increasing *ΔG*, albeit non-significantly (*R*_S_ = 0.259, *p* = 0.182).

## **DISCUSSION**

The aim of this study was to measure the effect of a contralateral broadband noise MOC elicitor on psychophysical estimates of cochlear gain using the TMC method (Nelson et al. 2001). The masker duration was shortened to minimize the possibility that the masker would itself elicit the MOC reflex in time to affect signal detection, and the elicitor was presented at a level of 54 dB SPL to avoid eliciting the MEM reflex. The signal level was fixed across the with- and without-elicitor conditions. The elicitor shifted the off-frequency TMC towards lower masking thresholds and lessened the slope and degree of non-linearity of the on-frequency TMC. The resulting change in the inferred cochlear IO function suggested that the elicitor had caused a reduction in cochlear gain. The amount of gain reduction was estimated by fitting the on- and off-frequency TMCs with a generic model of the cochlear tip and tail response IO functions. The model produced an excellent fit to both the with- and without-elicitor data. Across subjects, the without-elicitor gain was estimated as 23.9 dB, and the compression exponent as 0.18, in line with physiological estimates in primates (reviewed in Robles and Ruggero 2001). The elicitor-induced gain reduction was estimated as 4.4 dB, equivalent to 18.4 % of the without-elicitor gain. This corresponds well with the suppression of auditory nerve responses by moderate-level contralateral sound in animals (Warren and Liberman 1989). As a result, the compression exponent increased by 33 % from 0.18 to 0.27.

### Inter-Individual Variability in TMCs

The individual without-elicitor TMCs showed considerable variability with respect to the effects of the masker-signal gap and masking condition (on/off). This is consistent with previous results (Rosengard et al. 2005; Poling et al. 2012). Jennings et al. (2014) simulated a large set of individual TMCs using a mechanistic model of the auditory periphery proposed by Zilany et al. (2009). Their simulations suggested that detection efficiency was the primary cause of inter-individual variability in TMCs in normal-hearing subjects. In the current phenomenological model, detection efficiency is represented by the threshold signal-to-masker response ratio, *k*. In addition to *k*, the current model also contained parameters for the maximum cochlear gain (*G*_max_), the slope and position of the compressive region in the cochlear IO function (*c*, BP_ctr_), the decay rate of the masker effect over time (*μ*), and any passive attenuation of the off-frequency masker response (*P*). In the modeling section (“Cochlear IO Function Model” section), we showed that variation in each of these model parameters creates a distinct pattern of variation in the positions and shapes of the predicted TMCs (see Fig. 5). Variation in the threshold signal-to-masker ratio, *k* (Fig. 5D), causes the on- and off-frequency TMCs to shift along the abscissa by the same amount, leaving their slopes and relative positions unchanged. This does not explain the significant inter-individual differences in the average and relative slopes, and the relative positions along the ordinate, of the on- and off-frequency TMCs observed in the current study. This suggests that subjects with normal audiometric thresholds can exhibit considerable variability, not only in detection efficiency, but also in key physiological factors determining the cochlear IO function shape (*G*_max_), frequency tuning (*P*), and persistence of masking (*μ*).

The estimated elicitor-induced reduction in cochlear gain (*ΔG*) also showed a great deal of inter-individual variability, ranging from −3.8 % (gain enhancement) to 100 % of the without-elicitor gain in different subjects. At the same time, *ΔG* showed a high degree of repeatability across measurement sessions, indicating that the variability reflects systematic inter-individual differences rather than random measurement error.

### Comparison with Contralateral Elicitor Effects on Psychophysical Tuning Curves

A number of previous studies have measured contralateral MOC elicitor effects on psychophysical (frequency) tuning curves (PTCs; Kawase et al. 2000; Quaranta et al. 2005; Vinay and Moore 2008; Aguilar et al. 2013; Wicher 2013; Wicher and Moore 2014). PTCs measure the level of a variable-frequency masker needed to just mask a low-level, fixed-frequency signal. They are a popular measure of auditory frequency selectivity in humans. The previous studies have generally tended to find that the elicitor caused a reduction in masking threshold at masker frequencies remote from, but not at or close to, the signal frequency, leading to a broadening of the PTCs (indicative of lesser frequency selectivity). This is consistent with the current finding that, at the shortest masker-signal gap, the elicitor caused a reduction in the off-frequency, but not on-frequency, masking threshold. The previous studies that used a 2-kHz signal and a broadband noise elicitor like the current study (Kawase et al. 2000; Wicher and Moore 2014) found an average reduction in the off-frequency masking threshold of around 3–5 dB, consistent with the average 4.4-dB reduction in cochlear gain found in the current study. Both previous studies measured the elicitor effect on distortion product OAEs (DPOAEs). In one study (Kawase et al. 2000), the across-subject correlation between the psychophysical and OAE elicitor effects was significant, but in the other study (Wicher and Moore 2014), the correlation was non-significant as in the current study.

### Comparison with Ipsilateral Elicitor Effects

**FIG. 9:**
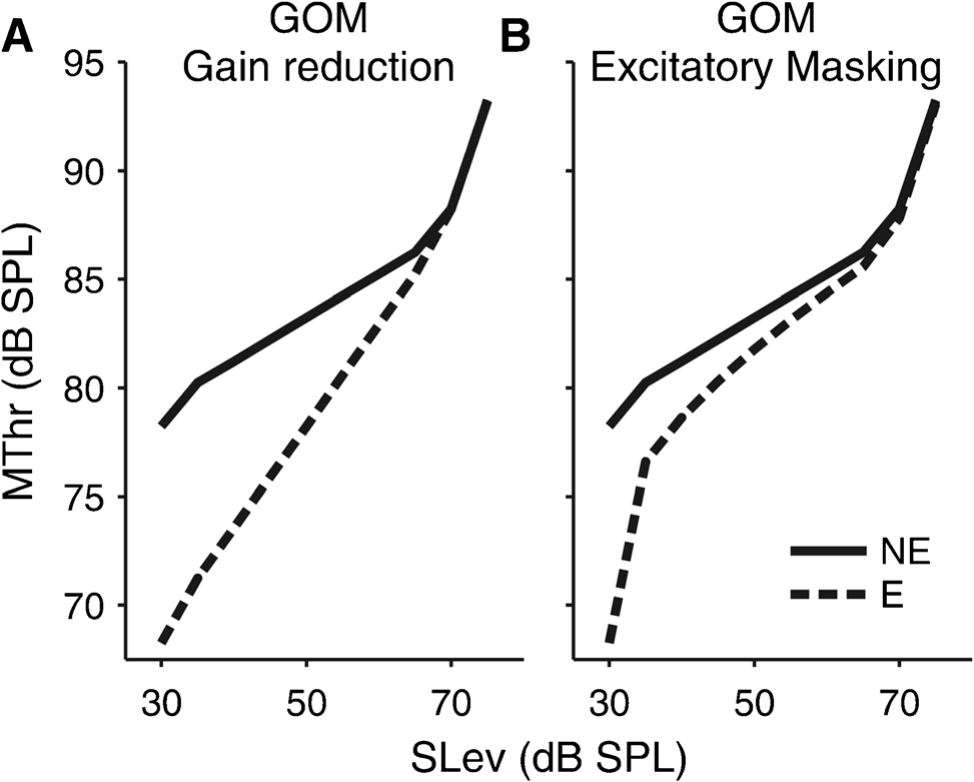
Predicted effects of elicitor-induced gain reduction **(A)** and direct excitatory masking on the off-frequency growth of masking (GOM) function. The *solid lines* show the without-elicitor functions (*NE*) and the *dashed lines* the with-elicitor functions (*E*; legend in **B**). The gain reduction and masking effects were set to cause the same amount of change in masking threshold (MThr) at the lowest signal level (SLev). The GOM functions were modelled using the same cochlear IO function model as used to model the TMCs shown in Figure 2 (*G*_max_ = 30 dB, *c* = 0.2, BP_1_ = 28.125 dB SPL, *k* = 1.5, and *P* = 20 dB).

A number of previous studies have measured ipsilateral MOC elicitor effects on psychophysical measures of cochlear gain and compression (Krull and Strickland 2008; Jennings et al. 2009; Roverud and Strickland 2010; Yasin et al. 2014). The elicitors were presented prior to the masker and signal (often referred to as “precursor”) to avoid direct acoustic interactions. In the studies by Strickland and colleagues (Krull and Strickland 2008; Jennings et al. 2009; Roverud and Strickland 2010), the signal level was fixed across the with- and without-elicitor conditions as in the current study, and cochlear gain and compression were measured using the growth of masking (GOM) method, which varies the signal or masker level rather than the masker-signal gap. The advantage is that the cochlear IO function can be inferred directly from the off-frequency GOM function (the function relating the off-frequency masking threshold with the signal level), removing the need for the on-frequency condition. Arguably, however, it is harder to distinguish whether the precursor effect is due to gain reduction or excitatory masking, because both effects cause the lower leg of the inferred IO function to shift downwards (towards lower masking thresholds; Fig. 9; compare Fig. 2B, D for the corresponding effects on the TMC-based IO function). Using sinusoidal precursors at the signal frequency, Strickland and colleagues found considerably larger gain reductions (of the order of 12 dB) than that found in the current study (4.4 dB) using a broadband noise elicitor. This contrasts with previous OAE suppression data, which have shown similar-sized (at 0.5 kHz) or smaller (at 1 and 4 kHz) MOC effects for ipsilateral narrowband than contralateral broadband noise elicitors (measured over the same post-elicitor time window; Lilaonitkul and Guinan 2009a), and even smaller effects for ipsilateral sinusoidal elicitors (Lilaonitkul and Guinan 2012). It is possible, however, that at least part of the psychophysical precursor effects reported by Strickland and colleagues were caused by excitatory masking.

Yasin et al. (2014) controlled for excitatory masking by adjusting the signal level in the with-elicitor condition so that the signal was perceived at the same sensation level as in the without-elicitor condition. They used the new fixed-duration masking curve (FDMC) method for measuring cochlear gain and compression (Yasin et al. 2013), which is similar to the TMC method except that it varies the relative masker and signal durations (with the overall duration fixed) rather than the masker-signal gap. Using a band-pass noise precursor centered on the signal frequency, Yasin et al. (2014) found even greater gain reductions than Strickland and colleagues (≥16 dB), even when the precursor level was very low (40 dB SPL). This contrasts with OAE suppression results by Guinan et al. (2003), who found little or no evidence of MOC effects at a 40-dB SPL elicitor level, irrespective of the type of elicitor sound used (tone pips, clicks, sinusoids, broadband noise). The results by Guinan et al. suggest that ipsilateral precursor effects may, at least in part, be caused by non-efferent processes intrinsic to the cochlea. Evidence for intrinsic cochlear effects is provided by adaptation of DPOAEs (Kujawa et al. 1995; Lowe and Robertson 1995; Liberman et al. 1996). It has been argued that intrinsic cochlear effects may be caused by local activation of non-efferent synapses on outer hair cells (OHCs), which may elicit the same OHC processes as are elicited by MOC efferents, and the effects of which should thus decay with the same time constants (Guinan et al. 2003).

### Implications for Absolute Threshold

The contralateral elicitor increased the quiet (“absolute”) threshold of the signal by less 1 dB (0.78 dB on average; up to 2.36 dB in individuals). This is consistent with recent results by Aguilar et al. (2015). At the same time, the elicitor had a significant effect on the masking thresholds (the maximum change in masking threshold across conditions was 4.8 dB on average and 17.6 dB in individuals). The masking threshold changes suggested a change in cochlear gain by an average of 4.4 dB, more than five times the elicitor-induced change in the signal quiet threshold (4.4/0.78 = 5.64). Thus, for every 1 dB of elicitor-induced reduction in cochlear gain, the signal quiet threshold increased by only around 0.2 dB (1/5.64 = 0.18 dB/dB). This finding is independently supported by the observation that, across subjects, the signal quiet threshold without the elicitor increased by only 0.21 dB per 1 dB decrease in individual without-elicitor cochlear gain. This suggests that, at least in normal-hearing subjects, absolute threshold is only weakly sensitive to cochlear gain, and thus that a significant proportion of the internal noise determining absolute threshold is, like the driven response, subject to cochlear gain (i.e., occurs at or before the stage of active cochlear amplification). This may explain the apparent contradiction between the findings that contralateral sounds cause little masking, particularly when the masker frequency is different from the signal frequency (reviewed in Mills et al. 1996), but significant suppression of driven cochlear responses (Puria et al. 1996).

### Comparison with OAE Suppression Data

The current study also measured contralateral suppression of CEOAEs, using the same elicitor and group of subjects as for the TMC measurements. The elicitor caused a general reduction in CEOAE amplitude, supporting the notion that the elicitor effect on the TMCs reflected a reduction in cochlear gain as a result of MOC activation. However, for the CEOAEs, the amount of reduction depended on the order in which the with- and without-elicitor conditions were measured: a significant reduction was only observed when the with- and without-elicitor conditions were interleaved within the same session, and not when they were measured separately. This order effect may be related to a similar order effect found by Micheyl and Collet (1996) on the correlation between contralateral OAE suppression and elicitor-induced improvement in the signal-in-noise detection. The fact that the order effect was specific to the OAEs (no order effect was found in the TMCs) suggests that it is unrelated to MOC-induced reduction in the cochlear gain. This is supported by the finding that individual elicitor-induced change in CEOAE amplitude was significantly correlated between the interleaved and separate sessions.

Whilst there was a tendency for individual elicitor-induced CEOAE suppression (ΔCEOAEn) to increase with cochlear gain reduction derived from the TMCs (*ΔG*), the correlation was non-significant. A similar finding was obtained by Wicher and Moore (2014; but see Kawase et al. 2000). It is likely that this was in part due to underpowering (the correlation between the without-elicitor gain and compression exponent was also non-significant) and in part due to systematic differences between the TMC and OAE measurements. The OAE measurements used broadband clicks as probe sounds and may thus reflect MOC feedback at a different cochlear place than the TMC measurements, which used 2-kHz sinusoids. Moreover, the clicks may have caused some MOC activation themselves, which may have diminished the elicitor-induced OAE suppression (Guinan et al. 2003). Previous findings of attentional influences on MOC feedback (Giraud et al. 1995; Maison et al. 2001; de Boer and Thornton 2007) suggest that the differences in task demand between the TMC and OAE measurements (no task was performed during the OAE measurements) may also have contributed to decorrelating their results. Finally, there is evidence suggesting that the MOC reflex is influenced by auditory learning (de Boer and Thornton 2008). This means that the relationship between the TMC and OAE results may have been different, had the OAEs been measured before, rather than after, the TMCs.

CEOAEs belong to a class of OAEs referred to as “place-fixed” emissions (Kemp 1986). The mechanisms by which this class of OAEs are generated and propagate back from their cochlear origin remain unresolved (Shera and Guinan 1999; Knight and Kemp 2000; Reichenbach et al. 2012), and so, the relationship between MOC-induced CEOAE suppression and reduction in cochlear gain remains unclear. To overcome this problem, Veuillet et al. (1991) measured contralateral CEOAE suppression as a function of probe level and derived the reduction in probe level that would produce an equivalent reduction in CEOAE amplitude to that produced by the elicitor (referred to as “effective attenuation,” Puria et al. 1996). Using a similar elicitor sound as used here (broadband noise at 50 dB SPL), they found an effective attenuation of 3.8 dB on average, remarkably similar to the average reduction in the cochlear gain (4.4 dB) found here based on the TMCs.

This suggests that MOC-induced amplitude suppression of place-fixed OAEs, transformed into effective attenuation, may show good correspondence with MOC-induced reduction in cochlear gain measured psychophysically. Testing this would resolve whether the discrepancies between the existing OAE and animal results (see “Introduction” section) reflect true species differences or are related to differences in measurement technique. For instance, the finding that contralateral elicitor effects on place-fixed OAEs, including spontaneous OAEs (SOAEs; Shera 2003), are strongest for probe (SOAE) frequencies below 3–6 kHz and for elicitor frequencies between 0.5 and 2 kHz (Lilaonitkul and Guinan 2012; Zhao and Dhar 2012) may be related to the mechanism by which place-fixed OAEs are generated. It has been suggested that place-fixed OAEs are generated by back-reflection of forward-travelling waves on the basilar membrane from random mechanical irregularities (Shera and Guinan 1999). The frequency dependence of contralateral suppression effects on place-fixed OAEs may thus reflect the density of irregularities along the cochlear length. Alternatively, it may be related to the cochlear amplification mechanism. OHC electromotility pulls and pushes the basilar membrane towards and away from the reticular lamina (at the top of the OHCs). This is assumed to amplify the basilar membrane motion (Robles and Ruggero 2001). However, recent experimental and modeling results suggest that the active OHC forces may independently amplify the motion of the reticular lamina (Chen et al. 2011; Zha et al. 2012), and may even become decoupled from the basilar membrane motion (Reichenbach and Hudspeth 2010). If place-fixed OAEs represent back-reflected waves on the basilar membrane, their sensitivity to MOC suppression should depend on the degree of this decoupling, which may be frequency-dependent (Reichenbach and Hudspeth 2010).

## **CONCLUSIONS**

The current results suggest that the perceptual TMC method for measuring cochlear gain and compression is also suitable for measuring contralateral MOC-induced reduction in cochlear gain. Here, the on- and off-frequency TMCs were fitted separately, before combining to infer the cochlear IO function. This makes it easier to estimate cochlear gain and compression in individual subjects, where inferred IO functions can sometimes be non-monotonic. Our results suggest that a contralateral broadband noise elicitor with a moderate level can reduce cochlear gain by a considerable proportion. The effect was highly repeatable within subjects, but showed considerable variability across subjects. Inter-individual variability in MOC reflex strength may contribute to inter-individual variability in susceptibility to hearing loss through sound exposure (Maison and Liberman 2000; Maison et al. 2013).
